# Large-Scale Phylogenomics of the *Lactobacillus casei* Group Highlights Taxonomic Inconsistencies and Reveals Novel Clade-Associated Features

**DOI:** 10.1128/mSystems.00061-17

**Published:** 2017-08-22

**Authors:** Sander Wuyts, Stijn Wittouck, Ilke De Boeck, Camille N. Allonsius, Edoardo Pasolli, Nicola Segata, Sarah Lebeer

**Affiliations:** a Research Group Environmental Ecology and Applied Microbiology, Department of Bioscience Engineering, University of Antwerp, Antwerp, Belgium; b Centre for Integrative Biology, University of Trento, Trento, Italy; University of California, San Diego

**Keywords:** *Lactobacillus casei* group, accessory Sec system, catalase, comparative genomics, phylogenomics

## Abstract

Although the genotypic and phenotypic properties of the *Lactobacillus casei* group have been studied extensively, the taxonomic structure has been the subject of debate for a long time. Here, we performed a large-scale comparative analysis by using 183 publicly available genomes supplemented with a *Lactobacillus* strain isolated from the human upper respiratory tract. On the basis of this analysis, we identified inconsistencies in the taxonomy and reclassified all of the genomes according to their most closely related type strains. This led to the identification of a catalase-encoding gene in all 10 *L. casei sensu stricto* strains, making it the first described catalase-positive species in the *Lactobacillus* genus. Moreover, we found that 6 of 10 *L. casei* genomes contained a SecA2/SecY2 gene cluster with two putative glycosylated surface adhesin proteins. Altogether, our results highlight current inconsistencies in the taxonomy of the *L. casei* group and reveal new clade-associated functional features.

**IMPORTANCE** The closely related species of the *Lactobacillus casei* group are extensively studied because of their applications in food fermentations and as probiotics. Our results show that many strains in this group are incorrectly classified and that reclassifying them to their most closely related species type strain improves the functional predictive power of their taxonomy. In addition, our findings may spark increased interest in the *L. casei* species. We find that after reclassification, only 10 genomes remain classified as *L. casei*. These strains show some interesting properties. First, they all appear to be catalase positive. This suggests that they have increased oxidative stress resistance. Second, we isolated an *L. casei* strain from the human upper respiratory tract and discovered that it and multiple other *L. casei* strains harbor one or even two large, glycosylated putative surface adhesins. This might inspire further exploration of this species as a potential probiotic organism.

## Abstract (precis)

The closely related species of the *Lactobacillus casei* group are extensively studied because of their applications in food fermentations and as probiotics. Our results show that many strains in this group are incorrectly classified and that reclassifying them to their most closely related species type strain improves the functional predictive power of their taxonomy. In addition, our findings may spark increased interest in the *L. casei* species. We find that after reclassification, only 10 genomes remain classified as *L. casei*. These strains show some interesting properties. First, they all appear to be catalase positive. This suggests that they have increased oxidative stress resistance. Second, we isolated an *L. casei* strain from the human upper respiratory tract and discovered that it and multiple other *L. casei* strains harbor one or even two large, glycosylated putative surface adhesins. This might inspire further exploration of this species as a potential probiotic organism.

## INTRODUCTION

*Lactobacillus* is the largest genus of lactic acid bacteria, comprising >200 species (1). These species are naturally present on human and animal mucosal surfaces (e.g., the gastrointestinal and vaginal tracts) and in many food-related environments, including fruits, vegetables, wine, milk, and meat, where they can become dominant if able to ferment large doses of sugar with concomitant production of lactic acid and related metabolites. These bacteria are important model microorganisms for metabolic fermentation, cell wall biosynthesis, and microbe-host interaction studies. In addition, they are currently exploited in many biotechnical applications, e.g., as starter cultures, as probiotics, in the production of bioplastics, and as vaccine carriers, highlighting their high commercial value (1).

The *Lactobacillus casei* group, comprising the species *L. casei*, *Lactobacillus paracasei*, and *Lactobacillus rhamnosus*, is among the most economically interesting clades within the genus *Lactobacillus*. Commercially, these microbes are used in fermented dairy products or food supplements targeting the gastrointestinal tract (2–4) and the vaginal tract (5). Interest is also increasing in their application in other product formulations targeting different human and animal body niches. For example, an underexplored niche for topical application of probiotics is the (upper) respiratory tract and its related diseases (6).

Despite broad interest in the *L. casei* group, both the nomenclature and the classification of this group are subjects of discussion. This is reflected, for example, in the introduction of the related species *Lactobacillus zeae* in 1996 (7) and its subsequent rejection in 2008 (8). Furthermore, recent comparative genomic analyses showed that many strains currently classified as *L. casei* and *L. paracasei* are, in fact, members of the same species (9). In addition, many new isolates are classified as *L. casei*, while they are genetically more closely related to *L. paracasei* type strain ATCC 334 than to *L. casei* type strain ATCC 393 because of high heterogeneity in their 16S rRNA genes (10). Thus, many novel identifications are not in line with the current taxonomic classification (4, 8, 11). Multiple efforts have been made to facilitate the differentiation between *L. casei* group members on the basis of the use of PCR and/or DNA fingerprinting techniques (4, 10–13). However, with the price reduction of whole-genome sequencing and the rising availability of public genomes (210 *L. casei* group members as of February 2017), a more in-depth insight into the genetic differences and taxonomy of *L. casei* group members can be obtained by using computational comparative genomics.

In this study, we isolated a lactic acid bacterium from the respiratory tract, a rather unexpected niche for such a bacterium because it is not anaerobic or nutrient/sugar rich. On the basis of its genome sequence and a comparative genomic analysis using 183 publicly available *L. casei* group genome assemblies, we classified this strain as a member of the species *L. casei*, which—unexpectedly—turned out to be the smallest clade within the *L. casei* group. To our knowledge, this isolate, which we named *L. casei* AMBR2, is the first *L. casei* strain isolated from the upper respiratory tract. Using different comparative genomic approaches, we provide more insight into the genetic relationship of strains belonging to this group and use this information to further explore the functional potential of *L. casei* AMBR2, as well as the other *L. casei* group members.

## RESULTS

In this study, we considered the 183 publicly available genome assemblies for the *L. casei* group that passed strict quality control (N75 values of >10,000 bp and <500 undetermined bases per 100,000 bases), as reported in Table S1 in the supplemental material. Of these genomes, 92 were originally annotated as *L. rhamnosus*, 36 were *L. casei*, 38 were *L. paracasei*, and 2 were *L. zeae*. In addition to the public genomes classified as belonging to the *L. casei* group, we screened all of the unclassified *Lactobacillus* genomes (categorized as *Lactobacillus* sp. in the NCBI database) for *L. casei* group members by comparing their 16S rRNA gene sequences to a filtered version of the RDP database (v11) (14). This resulted in an additional 15 genomes. Furthermore, one newly sequenced *L. casei* strain (AMBR2), which we isolated from a human upper respiratory tract, was added to the analysis. This resulted in a total of 184 *L. casei* group strains studied, thereby significantly improving the number of genomic assemblies studied since the latest comparative genomics study, where only 10 (2) or 34 (9) genomes were used.

10.1128/mSystems.00061-17.7 TABLE S1  Overview of all of the publicly available *L. casei* group genomes used in this study. If available, the strain name is shown in the second column. Download TABLE S1, CSV file, 0.01 MB. Copyright © 2017 Wuyts et al. 2017 Wuyts et al. This content is distributed under the terms of the Creative Commons Attribution 4.0 International license.

### Identification of three clades within the *L. casei* group on the basis of GC content, phylogeny, and pairwise comparisons.

**FIG 1:**
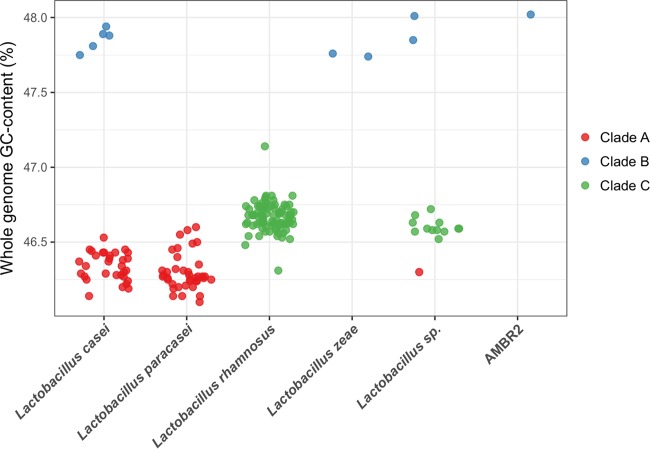
GC contents of all of the genomes analyzed in this study. Genomes are grouped according to their species annotation in the NCBI database (except for upper respiratory tract isolate *L. casei* AMBR2) and colored by the phylogenetic clade they belong to, as defined in Fig. 2.

The GC contents of the species *L. paracasei*, *L. rhamnosus*, and *L. zeae* show low intraspecies variation, with respective average values of 46.3, 46.7, and 47.8% (Fig. 1), whereas larger interspecies diversity is observed. In contrast, *L. casei* genomes can be divided into two groups, a large group showing a GC content in the range of that of *L. paracasei* (46.10 to 46.60%) and a small group of five genomes showing a much greater GC content, similar to that of the *L. zeae* genomes (47.74 to 47.76%). As for the unclassified assemblies (categorized as *Lactobacillus* sp.), two genomes show a GC content as great as that of the *L. zeae* genomes, while the rest of them are within the *L. rhamnosus-L. paracasei* range. The genome sequence of our new isolate, which we designated *L. casei* AMBR2, shows a GC content similar to that of the *L. zeae* genomes.

**FIG 2:**
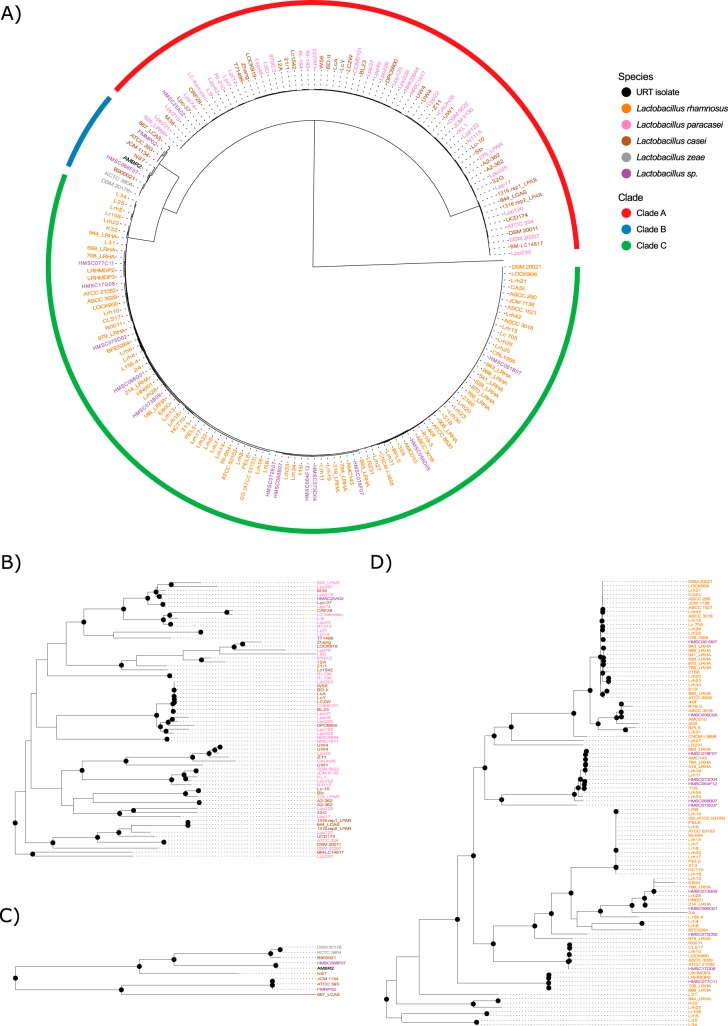
Phylogenetic trees of the whole *L. casei* group and the individual clades. (A) Phylogenetic tree constructed of all 184 genome assemblies of the *L. casei* group by using 776 single-copy marker genes identified by roary (15) with *L. nasuensis* JCM 17158 as the outgroup. Colors show NCBI database classifications. URT, upper respiratory tract. (B) Subtree of clade A. (C) Subtree of clade B. (D) Subtree of clade C. Strong clades (bootstrap support of >70) are indicated by black dots.

To study the genetic relatedness of the genomic assemblies, we constructed a high-quality maximum-likelihood phylogenetic tree of the *L. casei* group by using 776 conserved single-copy marker genes. These marker genes were identified with roary (15) and showed at least 70% blastp identity and were present in at least 96% of the genomes studied. In addition, the genome of *Lactobacillus nasuensis* JCM 17158 (GCA_001434705) was added to the alignment to serve as an outgroup. This strain was chosen because it had the best-quality assembly of three strains that are closely related to the *L. casei* group (1). The resulting tree is shown in Fig. 2A. The tree structure reveals three separate clades within the *L. casei* group, with very small branch lengths within each clade in comparison to the branch lengths between the clades. Clade A contains the majority of the *L. casei* genomes and all of the *L. paracasei* genomes, as well as one unclassified *Lactobacillus* sp. assembly. The smallest clade (B), contains the two *L. zeae* genomes, five *L. casei* genomes (including *L. casei* type strain ATCC 393), two unclassified lactobacilli, and our own upper respiratory tract isolate (*L. casei* AMBR2). Interestingly, these members of clade B are also those with an elevated GC content, as shown in Fig. 1. Finally, clade C consists of all *L. rhamnosus* genomes, as well as 12 *Lactobacillus* sp. genomes retrieved from the NCBI database as described above.

The phylogeny of the clade A subtree (Fig. 2B) shows that the genomes of *L. casei* and *L. paracasei* are completely intermixed in the tree. Together with the occurrence of *L. casei* genomes in clade B, this shows that these two species constitute paraphyletic taxa when the current species annotations are used. The two *L. zeae* genomes do cluster together in the subtree of clade B (Fig. 2C), but it should be noted that these genomes represent the same strain (DSM 20178 = KCTC 3804) independently sequenced. In contrast, *L. rhamnosus* does seem to be a monophyletic taxon according to this tree (Fig. 2D). Of note is the fact that multiple genomic assemblies seem to be identical on the basis of the sequences of all 776 single-copy marker genes. For example, there appear to be no fewer than 17 assemblies that show very high similarity to *L. rhamnosus* GG, a well-known probiotic strain (16), possibly indicating that the same strain was sequenced on multiple occasions.

**FIG 3:**
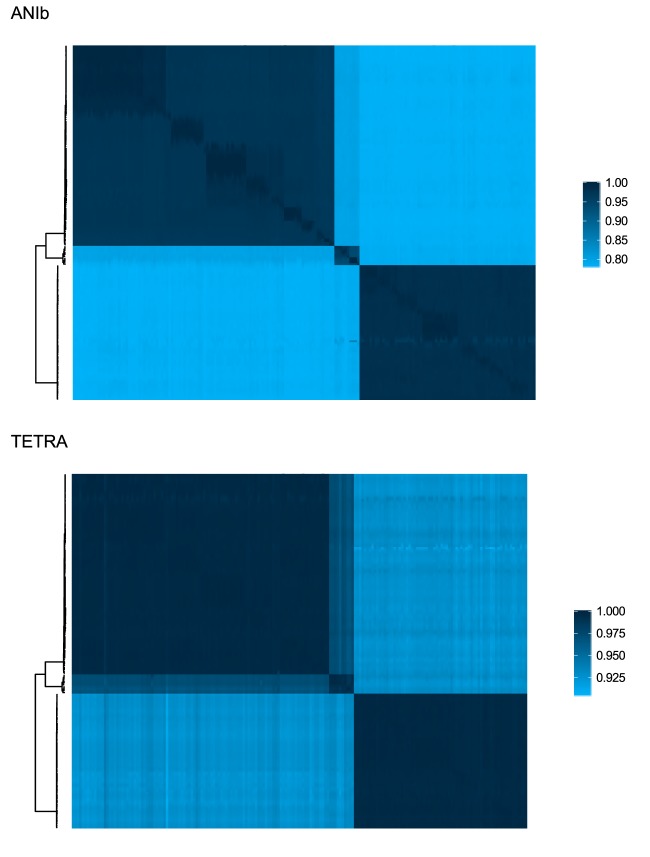
Pairwise ANIb and TETRA values for all genomes. The phylogenetic tree on the left is the same as that in Fig. 2A with the outgroup removed.

In the current era of whole-genome sequencing, pairwise genome comparison metrics such as average nucleotide identity (ANI) and tetranucleotide frequency (TETRA) are often used as an operational method to detect species boundaries. Figure 3 shows the ANIb (ANI calculated by using a blast implementation) and TETRA distances between all *L. casei* group genomes. Both distance metrics support the grouping of the genomes in the three clades defined by the phylogenetic tree in Fig. 2. For species delimitation, Richter et al. (17) advise the use of an ANI cutoff of 95 to 96%, although their tests show that values of 94 to 95% within a species are not uncommon. When comparing genomes belonging to different clades (e.g., a clade A genome and a clade B genome), we observed ANI values of ≤85.1%, indicating that each clade consists of one or more species that are distinct from the other clades. When comparing genomes that belong to the same clade (e.g., two clade A genomes), we observed ANI values of ≥96.1% in clade A, ≥93.6% in clade B, and ≥96.3% in clade C. These results suggest that clades A and C both consist of a single bacterial species. The minimal ANI value within clade B, however, is just below the species threshold of 94 to 95%, indicating that clade B might be considered one bacterial species with two subspecies or even two separate species. This conclusion is also supported by very high TETRA values within each clade; ≥0.9941 in clade A, ≥0.9872 in clade B, and ≥0.9882 in clade C.

### Gene content and predicted functional capacity support separation of the *L. casei* group in three clades.

In total, 521,567 genes were present in the genomes, with an average number of 2,828 (±141) genes per genome. These genes were clustered into 5,915 orthogroups by OrthoFinder (18), where an orthogroup is defined as the group of genes descended from a single gene in the most recent common ancestor of a group of species (18). Of these orthogroups, 1,814 were identified as core orthogroups and 4,101 were identified as accessory orthogroups (Table 1). When comparing clades A, B, and C identified above, there seems to be no large difference in the average number of genes per genome. Regarding the core orthogroups, clade C seems to have the largest number (2,133), while clades A and B were found to have exactly the same number of core orthogroups (1,924). Finally, clade B showed the lowest number of accessory orthogroups, probably because of the lower number of available genomic assemblies.

**TABLE 1:** Overview of the gene content distribution in the *L. casei* group^a^

Group	No. of genomes	Avg no. of genes/genome ± SD	Avg no. of orthogroups/genome ± SD	No. of core orthogroups	No. of accessory orthogroups
*L. casei* group	184	2,827 ± 141	2,654 ± 92	1,814	4,101
Clade A	70	2,897 ± 148	2,696 ± 106	1,924	2,866
Clade B	10	2,847 ± 111	2,615 ± 96	1,924	1,576
Clade C	104	2,780 ± 119	2,629 ± 68	2,133	2,363

a Gene content metrics were calculated for the *L. casei* group as a whole, as well as for the three clades defined by the phylogenetic tree. A core orthogroup is defined as an orthogroup present in >95% of the genomes.

**FIG 4:**
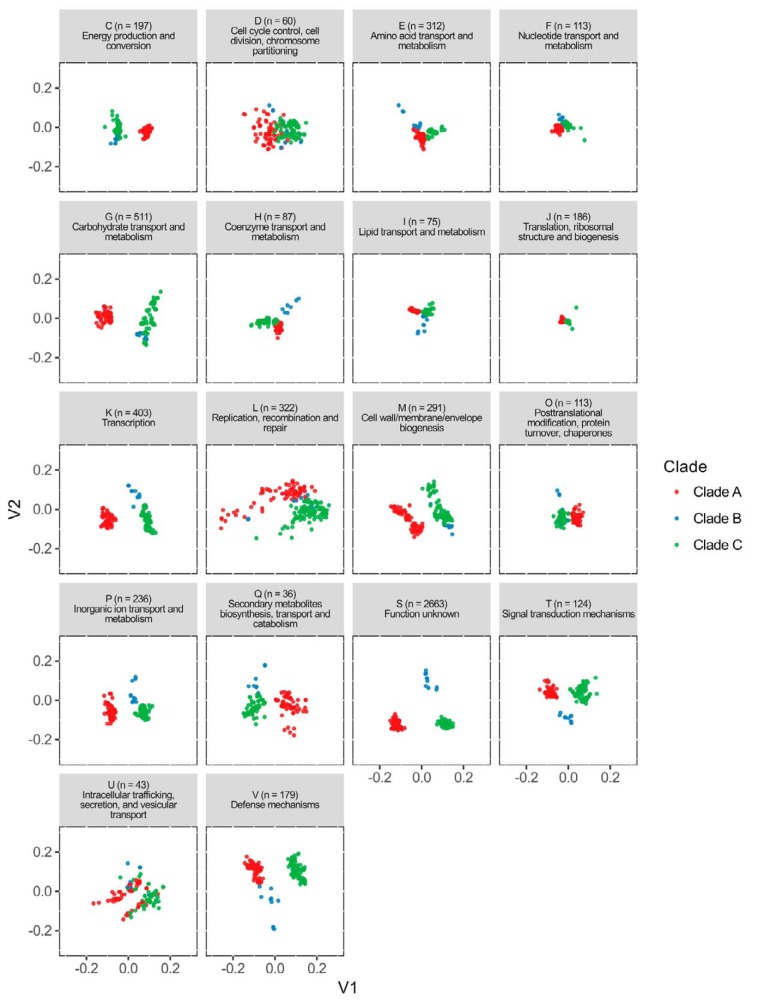
PCoA of predicted functional capacity per clade based on mapping of all orthogroups to the eggNOG database (v4.5) (19). Each letter represents a different functional category, as defined above each plot. Some orthogroups mapped to multiple functional categories. The majority of the orthogroups (2,662 of them) mapped to category S (function unknown).

To compare the overall functional capacity of the strains, we visualized the differences in gene content of the genomes for different functional categories of genes. This was done by mapping the orthogroups to the eggNOG database (19). In total, between 2,332 and 2,902 genes per genome had a match within 18 functional categories (Fig. 4). However, it should be noted that a great fraction of orthogroups (*n* = 2,662) was categorized as “function unknown” (S), showing the necessity for further experimental research in functional gene validation.

Figure 4 shows that all of the genomes studied have a rather overlapping orthogroup composition in categories D and J, respectively, representing cell cycle control, cell division, chromosome partitioning and translation, ribosomal structure, and biogenesis functions, two very fundamental categories in which no significant difference between closely related species is expected. Members of clades B and C cluster separately from members of clade A for the functions energy production and conservation, carbohydrate metabolism, cell envelope biogenesis, and posttranslational modification and chaperons (categories C, G, M and O). In contrast to these are categories E and V, representing amino acid transport and metabolism and defense mechanisms, where the opposite grouping was observed since clade B showed greater similarity to clade A. Interestingly, in at least 6 of 18 categories (H, I, K, P, Q, and T), a clear separation between the members of the phylogenetically different clades could be observed, indicating that these clades possess different functional capacities and highlighting the great potential for further investigation in future work. As a side note, no systematic differences between the clades in the total gene counts of the functional groups were observed (see Fig. S1), so we could exclude this as a possible explanation for why we see cladewise grouping in our principal-coordinate analysis (PCoA) plots.

10.1128/mSystems.00061-17.3 FIG S1  Gene counts per functional category. The number of genes per functional category in each genome was determined. Download FIG S1, EPS file, 0.1 MB. Copyright © 2017 Wuyts et al. 2017 Wuyts et al. This content is distributed under the terms of the Creative Commons Attribution 4.0 International license.

### Genetic potential for oxidative stress resistance is a clear discriminative feature of the clades within the *L. casei* group.

The three phylogenetic clades differ in functional potential on the basis of the generic orthogroup analysis presented. Therefore, we further explored their functional potential by means of a more in-depth interest-driven study of two industrially relevant properties of lactic acid bacteria, oxidative stress resistance and glycosylation potential.

Being able to cope with the presence of reactive oxygen species is an important feature for the industrial use, as well as the niche adaptation, of lactobacilli. Since *L. casei* AMBR2, which was isolated from the upper respiratory tract of a healthy person, a typical oxygen-rich niche, and documented oxidative-stress-resistant strain *L. casei* N87 (20) both cluster within smaller clade B of the *L. casei* group, we evaluated different oxidative stress resistance mechanisms of clade B in relation to all *L. casei* group members.

Catalase, which catalyzes the decomposition of H_2_O_2_ to H_2_O and O_2_, plays an important role in protecting cells against oxidative stress. While the *Lactobacillus* genus is defined as catalase negative, recent studies have shown catalase activity in several strains, including respiration-competent strain *L. casei* N87 (21), but without linking the presence of these catalase genes with the underlying phylogeny. Therefore, the presence of catalase genes was evaluated here in relation to the newly described phylogenetic structure of the *L. casei* group. Interestingly, open reading frames annotated as catalase genes were identified only in strains belonging to clade B and in a single genome of clade C (*L. rhamnosus* CRL1505; GCA_000414365). Mapping to Pfam families PF00199 and PF05067 with HMMER (22) resulted in the identification of two different catalase types, one annotated as a heme-dependent catalase (487 amino acids) and the other as a manganese-dependent catalase (269 amino acids). The heme-dependent catalase gene was found in all 10 genome assemblies of clade B, while the manganese-dependent catalase gene was present in only 7 of 10 clade B genomes. We could experimentally confirm that only the strains of clade B tested—particularly strain AMBR2—were positive for catalase activity by a standard microbiology lab test (Table S2).

10.1128/mSystems.00061-17.8 TABLE S2  Overview of the catalase activities of 12 strains tested after 24 h of growth under four different conditions. Production of catalase was assessed as described previously (see reference 1 in the Table S2 file). Briefly, 12 selected strains (4 per clade) were grown in Weissella medium broth (WMB) (see reference 2 in the Table S2 file) under different conditions, i.e., anaerobiosis (static cultivation in AnaeroCult jars [Merck Millipore, Overijse, Belgium] or AnaeroGen bags [Oxoid, Hampshire, United Kingdom] with or without supplementation with 2.5 µg/ml hemin [Sigma-Aldrich, Diegem, Belgium] and 1 µg/ml menaquinone [Sigma-Aldrich]) or aerobiosis (agitation on a rotary shaker at 150 rpm with or without respiration-promoting supplementation with hemin and menaquinone). After 24 h, the washed biomass derived from 1 ml of culture was resuspended in 100 µl of 3% (vol/vol) H_2_O_2_ (Carl Roth, Karlsruhe, Germany). Bubble or froth formation provided an indication of the presence of catalase activity. The conditions are abbreviated as follows: AN, anaerobiosis; AN+, anaerobiosis with addition of heme and menaquinone; AE, aerobiosis; AE+, aerobiosis with addition of heme and menaquinone. Download TABLE S2, DOCX file, 0.01 MB. Copyright © 2017 Wuyts et al. 2017 Wuyts et al. This content is distributed under the terms of the Creative Commons Attribution 4.0 International license.

The antioxidant superoxide dismutase (SOD), which is encoded by another gene important for oxidative stress resistance and scavenges O_2_^−^ into O_2_ and H_2_O_2_, was long believed to be absent from the *Lactobacillus* genus. However, genome analysis recently revealed the presence of SOD-encoding genes in some *L. casei* and *L. paracasei* strains (23), suggesting that two of the three *L. casei* group species could harbor SOD-encoding genes. To confirm this, we screened the whole *L. casei* group for SOD-encoding genes. Remarkably, SOD-encoding genes were present only in our newly assigned clade A strains (69 of 70 genomes) and thus in only one species instead of two. Interestingly, mapping to four different SOD Pfam families (PF00080, PF00081, PF02777, PF09055) with HMMER led to two different hits, one expected hit with PF00081 representing the gene for iron-manganese SOD (found in 69/70 clade A genomes) and one rather unexpected hit with the gene for copper SOD (found in 4/70 clade A genomes), which is the SOD most commonly used by eukaryotes. In general, these results show that because of the new phylogenetic structure of the *L. casei* group presented in this paper, the presence of SOD-encoding genes seems to be a unique property of clade A.

### Six clade B genomes encode a SecA2/SecY2 secretion system with two putative glycosylated surface adhesins as substrates.

Strain-specific glycosylation of surface molecules greatly affects interactions between *Lactobacillus* bacteria and their host cells. In addition, it confers interesting industrial properties, such as improved rheological and stress resistance properties (24, 25). Therefore, we subsequently scanned the genomes for clade-specific patterns in gene families encoding glycosyltransferases (GTs). We then designed a visualization approach that allowed us to better align these GTs and their positions in a selection of genomes (all genomes of clade B and closed genomes of clades A and C). These results are shown in Fig. S2 to S4, respectively.

10.1128/mSystems.00061-17.4 FIG S2  Positions of GT-encoding genes in the genomes of clade A. The *x* axis represents the coordinates of the genes in the genome. Contigs were ordered by mapping them to the available closed genome of *L. paracasei* ATCC 334. Download FIG S2, EPS file, 0.04 MB. Copyright © 2017 Wuyts et al. 2017 Wuyts et al. This content is distributed under the terms of the Creative Commons Attribution 4.0 International license.

10.1128/mSystems.00061-17.5 FIG S3  Positions of GT-encoding genes in the genomes of clade B. The *x* axis represents the coordinates of the genes in the genome. Contigs were ordered by mapping them to the contigs of *L. casei* AMBR2. Download FIG S3, EPS file, 0.04 MB. Copyright © 2017 Wuyts et al. 2017 Wuyts et al. This content is distributed under the terms of the Creative Commons Attribution 4.0 International license.

10.1128/mSystems.00061-17.6 FIG S4  Positions of GT-encoding genes in the genomes of clade C. The *x* axis represents the coordinates of the genes in the genome. Contigs were ordered by mapping them to the available closed genome of *L. rhamnosus* GG. Download FIG S4, EPS file, 0.03 MB. Copyright © 2017 Wuyts et al. 2017 Wuyts et al. This content is distributed under the terms of the Creative Commons Attribution 4.0 International license.

We found five different gene clusters that were enriched in GTs, where a gene cluster was considered “enriched” if it contained three or more GTs with ≤5 kb between two successive GTs in at least one genome. Two of these five clusters contained a homolog of a known priming GT and thus are probably responsible for the biosynthesis of heteropolymeric exopolysaccharides (EPS) or capsular polysaccharides (CPS). These two clusters were present in all of the genomes but varied strongly between strains in the quantity and type of associated GTs found; the minimum was one GT (always the priming GT homolog), and the maximum was five GTs in one cluster. The third cluster contained one GT2 and one GT83 (on the basis of CAZY family numbers) in its minimal form (clade B), while more copies of these two GT types were found in clades A and C. A fourth cluster was detected in some clade C genomes (one to three GTs) and in all clade B genomes (four GTs). The fifth cluster could be found in 6 of 10 clade B genomes and was absent from clades A and C. It was located directly upstream of the fourth cluster.

**FIG 5:**
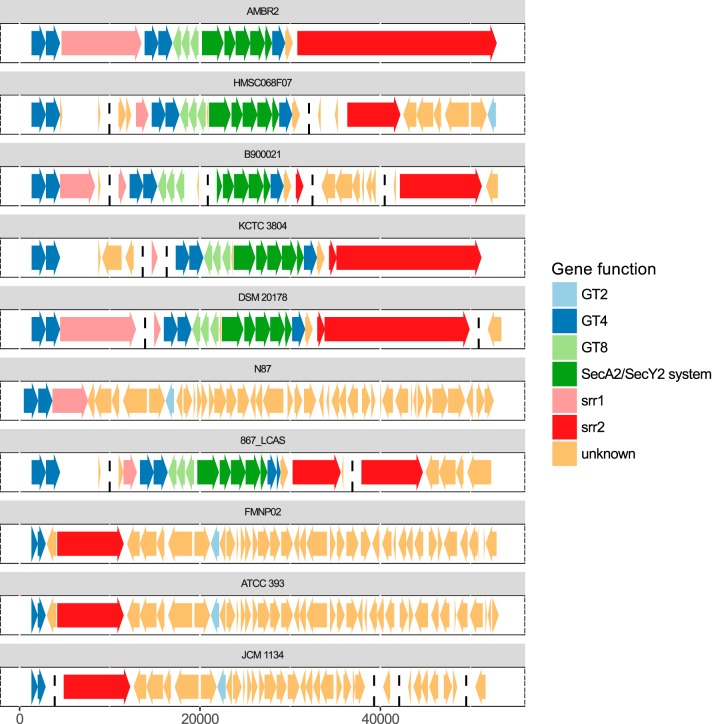
Gene content and order of the clade B-specific GT-rich gene cluster. The gene cluster is shown in all 10 clade B strains. Contigs were mapped to AMBR2; contig boundaries are indicated by broken vertical lines. Functional annotation was performed on the orthogroup level; multiple orthogroups can have the same function assigned to them. For example, the SecA2/SecY2 system consists of five orthogroups, SecA2, SecY2, Asp1, Asp2, and Asp3.

Because this fifth GT-rich gene cluster appeared to be a unique characteristic of clade B and was present in our isolate, *L. casei* AMBR2, we explored this cluster in more depth (Fig. 5). Of the six strains where the cluster was found, AMBR2 was the only one with an unfragmented genome sequence in this region. Therefore, we used this strain as a reference to describe the cluster. We defined the beginning and end of the cluster by considering the stretch of genes whose orthogroups were absent from clades A and C or at least less abundant than the orthogroups of the surrounding genes (see Table S3). In AMBR2, the cluster consisted of the following genes. The first is a long gene (8,868 bp) enriched in serine residues (15%), which we annotated as *srr1* (serine-rich repeat [Srr] gene 1). Directly downstream from that gene, we found two tandem GTs of the GT4 family, followed by three tandem GTs of the GT8 family located on the opposite strand. Furthermore, a SecA2/SecY2 secretion system, another GT4 gene, and a gene with an unknown function were found. The last is a second very long gene (22,113 bp; *srr2*) even more strongly enriched in serine residues than *srr1* (38%). The complete gene cluster, including the order of the genes, appeared to be fully conserved among the six strains that contained it. The cluster was absent from strain *L. casei* N87, except for the first part of *srr1*. In *Lactobacillus* sp. strain FMNP02, *L. casei* ATCC 393 (the *L. casei* type strain), and *L. casei* JCM 1134, only the last part of *srr2* was present.

10.1128/mSystems.00061-17.9 TABLE S3  Orthogroups in and around the clade B-specific GT-rich gene cluster. The distribution over the clades of the orthogroups in and around the GT-rich gene cluster specific to clade B is shown. Download TABLE S3, CSV file, 0 MB. Copyright © 2017 Wuyts et al. 2017 Wuyts et al. This content is distributed under the terms of the Creative Commons Attribution 4.0 International license.

The clade B-specific gene cluster described above contains two large genes that encode serine-rich proteins. These Srr proteins are often described as substrates for the accessory Sec (or SecA2/SecY2) system, which is responsible for the secretion of a single substrate (26). Therefore, we further investigated whether *srr1* and *srr2* could encode SecA2/SecY2 substrates. Unfortunately, we did not have the complete sequence of *srr1*. In AMBR2, the protein lacked its first 57 residues because of a dinucleotide deletion that caused a frameshift. In the other strains where the gene was present, it was truncated (N87) or its sequence was interrupted by a contig boundary (other strains). However, we could reconstruct the probable full sequence of the gene by looking at the alignment of the partial sequences (Text S1). From this reconstruction, it was clear that the protein contained both the KxYKxGKxW and the LPxTG motifs that are characteristic of a SecA2/SecY2 substrate (26), marking it as a highly probable accessory Sec system substrate and thus as a potential glycosylated surface adhesin.

10.1128/mSystems.00061-17.1 TEXT S1  Multiple-sequence alignment of *srr1* sequences. Download TEXT S1, TXT file, 0.03 MB. Copyright © 2017 Wuyts et al. 2017 Wuyts et al. This content is distributed under the terms of the Creative Commons Attribution 4.0 International license.

The *srr2* gene was present in its full form in AMBR2 (Text S2). In at least two strains, the first ∼280 amino acids of the protein, including the N-terminal signal sequence, were split off as an extra gene, also because of a frameshift mutation (a stretch of five A bases gained an additional A). In the other three strains, the sequence was split up because of contig boundaries. Interestingly, this protein also contained the KxYKxGKxW and LPxTG motifs, although the latter was a rare variant (LPQTS). This would mean that *srr2* codes for a second, different glycosylated surface adhesin secreted by the same accessory Sec system.

10.1128/mSystems.00061-17.2 TEXT S2  Multiple-sequence alignment of *srr2* sequences. Download TEXT S2, TXT file, 0.1 MB. Copyright © 2017 Wuyts et al. 2017 Wuyts et al. This content is distributed under the terms of the Creative Commons Attribution 4.0 International license.

In an attempt to identify the ligands of these two putative glycosylated surface adhesins, we performed a sequence homology search of the UniProt database. The best-scoring hit for Srr protein 1 was an uncharacterized protein from *Mycobacterium kansasii* that showed only 47% identity and 39% query coverage. The best-scoring hit for Srr2 was a “cell surface anchor protein” from *Streptococcus pneumoniae*, which also showed low identity and query coverage values (54 and 64%, respectively). Thus, we conclude that the two putative adhesins are as-yet-uncharacterized proteins that require further functional validation.

## DISCUSSION

In this study, the genome of *L. casei* AMBR2, an isolate from the upper respiratory tract, was sequenced and compared to all currently available high-quality genomic assemblies of the *L. casei* group, which comprises the closely related species *L. casei*, *L. paracasei*, *L. rhamnosus*, and *L. zeae*.

Here we show that the *L. casei* group consists of three different taxonomic clades on the basis of (i) differences in whole-genome GC content (Fig. 1), (ii) a phylogenetic tree constructed on the alignment of 776 single-copy marker genes (Fig. 2A), and (iii) pairwise ANI and TETRA values (Fig. 3). The branches of the phylogenetic tree that separate the three clades are well supported, but interestingly, a nonnegligible number of branches within the clades show low bootstrap support, indicating extensive horizontal gene transfer within the clades. We found that clade C represents the species *L. rhamnosus*, as it is uniquely made up of *L. rhamnosus* isolates, including the type strain *L. rhamnosus* DSM 20021. In contrast, both *L. casei* and *L. paracasei* isolates are found in clade A, which contains *L. paracasei* type strain ATCC 334. Because of the presence of this type strain, we suggest that all of the strains within clade A should be reclassified as *L. paracasei*, which is in line with the findings of Smokvina et al. (9). The third group, clade B, is much smaller than the other two clades (10 genomic assemblies) and consists of *L. casei*, *L. zeae*, and upper respiratory tract isolate *L. casei* AMBR2. Following the reclassification of *L. zeae* as *L. casei* (27), this group contains only *L. casei* strains, including *L. casei* type strain ATCC 393. Therefore, we propose that clade B represents the species *L. casei*. According to these results, only 5 of 36 genomes annotated as *L. casei* in the NCBI database are, in fact, genuine members of the species *L. casei*. The rest should be classified as *L. paracasei* instead, making *L. casei* the least sequenced species within the *L. casei* group. Of note is also the observation that *L. casei* is more closely related to *L. rhamnosus* than to *L. paracasei*, rendering the naming slightly confusing.

To our knowledge, this study is the first to perform an in-depth comparative genomics analysis of the *L. casei* group as a whole. We found the core genome of the whole group to be around 1,814 orthogroups, while the core genomes of reclassified clades A (*L. casei*), B (*L. paracasei*), and C (*L. rhamnosus*) contained around 1,924, 1,924, and 2,133 orthogroups, respectively (Table 1). The number of core orthogroups we found in the *L. casei* group is similar to that found by Smokvina et al. (9), who described a core genome of around 1,800 orthologous genes for 34 *L. casei* and *L. paracasei* genomes together. In clade C, we identified a slightly lower number of orthogroups than the 2,419 core genes described by Douillard et al. (28). Mapping of these orthogroups to the eggNOG database (v4.5) (19) revealed a difference in the predicted functional capacity of all three clades in at least 6 of 18 categories (Fig. 4). These results suggest that each phylogenetic clade could show unique functional properties previously overlooked because of taxonomic misclassifications. As an example, we worked out a comparative genomic analysis of two such functional properties, oxidative stress resistance and surface glycosylation potential. We show that both properties exhibit clade-specific gene distributions.

Of the three clades, B shows the largest intraclade variation. The lowest ANI value between two genomes in this clade was 93.6%, which is slightly lower than the cutoff normally used for bacterial species delimitation (17). In addition, clade B shows larger variation than the other clades in terms of their orthogroup content (see Fig. 4). Therefore, future work (including the isolation and sequencing of more clade B genomes) will indicate whether this clade should be split into more separate species.

In the past, several studies have successfully induced a more oxidative-stress-resistant phenotype in members of the *L. casei* group by means of heterologous expression of SOD and/or catalase (29–32), emphasizing the industrial importance of this phenotype. In this study, we evaluated the presence of oxidative-stress-related genes in the whole *L. casei* group. Surprisingly, we identified a SOD-encoding gene in all of the genomic assemblies of clade A. Apart from Chaillou et al. (33) and Liu et al. (34), who found the presence of a SOD-encoding gene in *Lactobacillus sakei* 23K and *L. casei* Lc18, respectively, to our knowledge, no other SOD-encoding gene has been described in the *Lactobacillus* genus. The translated SOD-encoding gene identified shows high similarity (99% identity, blastp) to the translated SOD-encoding gene from *L. casei* Lc18, as described by Liu et al. (34). This gene has been used for expression in *Escherichia coli*, which resulted in a small increase in scavenging activity, but the results were not convincing. We believe that further work in characterizing the activity of this SOD-encoding gene is necessary. Nevertheless, the presence of this gene is an interesting unique genomic property of clade A, representing the species *L. paracasei*, that was previously overlooked, possibly because of misclassification of the *L. casei* group members.

Catalase is another important driver in establishing an oxidative-stress-resistant phenotype. Although lactic acid bacteria are generally defined as catalase negative, catalase activity has been found in members of the genera *Lactobacillus*, *Pediococcus*, and *Leuconostoc* (35). For the genus *Lactobacillus* in particular, catalase genes and activity were reported and studied in *L. sakei* (29, 36), *Lactobacillus plantarum* (37–39), *L. casei* N87, and *L. zeae* (40). Here we show that all of the members of clade B in the *L. casei* group carry a gene for a heme-dependent catalase, making it the first described *Lactobacillus* species that is catalase positive. In addition, we identified a gene encoding a manganese-dependent catalase in 7 of 10 clade B members. In other words, the majority of the assemblies in this clade carry two different catalase-encoding genes. One member of this clade is *L. casei* N87, an oxygen-tolerant and respiratorily competent strain that has been extensively studied in the last few years (20, 21, 23, 41). These studies show that this strain possesses remarkably high oxidative resistance without the need for DNA cloning and thus support the fact that the predicted genes do function. This property makes the members of clade B, especially those carrying two catalase genes, of outstanding interest for industrial applications. Of interest is also the fact that strain AMBR2, which we isolated from the human nasopharyngeal niche, clusters within clade B and thus possibly harbors a very oxidant-resistant phenotype, as suggested here by the experimental observation of its catalase activity (Table S2).

The SecA2/SecY2 secretion system (or accessory Sec system) occurs in some Gram-positive bacteria in addition to the general SecA secretion system, which is universally present in bacteria. In each strain where it has been described, the accessory Sec system appears to be responsible for the secretion of a single substrate (26). This substrate differs between strains, but it is always a glycosylated surface adhesin belonging to a family of proteins (called Srr proteins) that can bind to a variety of ligands. The accessory Sec system and its substrates have been investigated primarily in Gram-positive pathogens such as *Streptococcus pneumoniae* and *Staphylococcus aureus* (26). In these species, virulence could be abolished by inactivating the secretion of the Srr protein. The accessory Sec system has also been found in some commensal Gram-positive bacteria, where it proved to be vital for successful host colonization. For example, Frese et al. (42) identified the SecA2/SecY2 system as the primary factor responsible for host-specific biofilm formation in *Lactobacillus reuteri*. Inactivation of the putative surface adhesin secreted by the system fully prevented host-specific biofilm formation. Since strain AMBR2 was isolated from the human upper respiratory tract, we hypothesize that one or both of its putative glycosylated surface adhesins are responsible for binding to a host ligand molecule in this niche, but this requires further experimental validation.

To our knowledge, this is the first time that two putative substrates for the same accessory Sec system have been identified within the same strain. However, even though both Srr proteins were present in some form in all six strains containing the accessory Sec system, we never observed them both in their fully intact form (i.e., including the N-terminal signal motif) within the same genome. In three strains, they might both be fully intact, but this was impossible to assess because of the quality of the assemblies. On three occasions, a frameshift mutation seemed to have occurred in one of the *srr* genes that cut off the N-terminal signal motif from the rest of the gene; once in *srr1* of AMBR2 and once in *srr2* of KCTC 3804 and DSM 20178 (or in their common ancestor). This observation might not be coincidental. It could be that the two surface adhesins are in competition with each other for secretion by the SecA2/SecY2 pathway. In the presence of a surface-attached ligand for only one of the adhesins, a short-term advantage might be gained by deactivating the other adhesin to adhere faster and better to the surface. This might constitute an interesting example of phase variation, i.e., the process of switching the expression of a gene on and off every couple of generations, leading to phenotype heterogeneity within a clonal bacterial population (43).

### Conclusions.

In this study, we sequenced a novel upper respiratory tract isolate, *L. casei* AMBR2, and studied its genome in relation to all of the currently available genomic assemblies of the members of the *L. casei* group. We found that the *L. casei* group harbors three different taxonomic clades by using a core genome phylogenetic tree, GC content analysis, and pairwise genome distances (ANIb and TETRA). On the basis of the presence of a different type strain in each of these clades, we propose that clade A represents the species *L. paracasei*, clade B represents the species *L. casei*, and clade C represents the species *L. rhamnosus*. Our study clearly shows that many *L. casei* strains are wrongly annotated in the NCBI database and should be reclassified as *L. paracasei*.

Reclassification of the *L. casei* group members led to the discovery of at least one catalase gene in all of the members of clade B, representing *L. casei*, making it the first described catalase-positive species in the whole *Lactobacillus* genus. In addition, we found that all *L. casei* group strains contain two putative EPS/CPS clusters and that six strains of clade B, among which is our isolate AMBR2, contain an accessory secretion system with two putative glycosylated surface adhesins as secreted substrates.

Finally, we propose the use of whole-genome ANI with respect to *L. casei* group type strains as an easy, computationally inexpensive metric to differentiate between the species *L. casei* and *L. paracasei*, on the condition that the genome has been sequenced. Alternatively, if sequencing of the 16S rRNA gene leads to the identification of a member of the species *L. casei* or *L. paracasei*, then we propose the use of the heme-dependent catalase gene or the SOD-encoding gene as a marker gene for the correct identification of these species.

## MATERIALS AND METHODS

### Sequencing and downloading of publicly available assemblies.

Whole-genome sequencing of *L. casei* AMBR2 was performed with the Nextera XT DNA Sample Preparation kit (Illumina, San Diego, CA), and sequencing with the Illumina MiSeq platform with 2 × 300 cycles at the Center of Medical Genetics Antwerp (University of Antwerp). Assembly was performed with SPAdes 3.8.0 (44).

All genomic assemblies classified as *L. casei*, *L. paracasei*, *L. rhamnosus*, and *L. zeae* (210 in total) were downloaded from the NCBI database on 19 February 2017 with in-house scripts. In addition, all unclassified *Lactobacillus* assemblies (annotated as *Lactobacillus* sp.; 28 in total) were screened for *L. casei* group members by blast searching (45) them against a filtered RDP database (v11) (14) containing only good-quality *Lactobacillus* 16S rRNA gene sequences longer than 1,200 nucleotides from cultured isolates. This resulted in 15 additional assemblies that were subjected to quality control.

### Quality control and whole-genome GC content.

The quality of the genomic assemblies was evaluated by using the output generated by Quast 4.3 (46). After visualization of different quality control parameters, genomes with N75 values of <10,000 bp and >500 undetermined bases per 100,000 bases were discarded. Subsequently, one genomic assembly (GCA_001063295) was removed, as it had a genome size of 5.8 Mbp and was identified as a hybrid assembly. The whole-genome GC content was calculated with Quast 4.3 (46) and infoseq from EMBOSS 6.6.0.0 (47), respectively. Visualization was done in R with ggplot2 (48).

### Gene prediction and annotation.

A custom genus-specific BLAST database was created by using all of the complete *Lactobacillus* genomes found in the NCBI database. This database was used in Prokka (49) with the −usegenus option to predict genes and annotate all genomic assemblies.

### ANIb and TETRA.

All pairwise ANIb and TETRA values were calculated with the Python pyani package (50) and visualized with the R package ggtree (51).

### Phylogenetic tree inference.

The generation of an alignment of the DNA sequences of a set of single-copy core genes was performed with roary (15) by using a minimum blastp identity of 70% and a threshold of 96% as the percentage of isolates a gene must be in to be defined as a conserved marker gene. Marker genes were translated and compared with a BLAST database of the outgroup GCA_001434705 (*L. nasuensis* JCM 17158) genome proteins. The DNA sequences of all hits with a coverage of >75% and an identity of >50% were added to the alignment by using in-house scripts. This alignment was used in RAxML 8.2.9 (52) to build a maximum-likelihood phylogenetic tree with the −a option, which combines a rapid bootstrap algorithm with an extensive search of the tree space starting from multiple different starting trees. The tree and subtrees were plotted with the R package ggtree (51).

### Orthogroup inference and gene content analysis.

Orthogroups were inferred with OrthoFinder (18) and analyzed in R by using in-house scripts. A core orthogroup is defined as an orthogroup present in >95% of the genomes. All representative sequences of all clade-specific core genes were scanned against a hidden Markov model (HMM) database of all *Bacillus* eggNOG (v4.5) (19) profile HMMs by using HMMER version 3.1b1 (22). Distance matrices based on orthogroup abundance profiles were calculated with the R package vegan (53) and visualized with ggplot2 (48).

The visualization of gene content per functional category was constructed in two steps. First, we mapped all orthogroups to gene families in the eggNOG database. All of the families in this database have functional categories assigned to them, which allowed us to split the orthogroups into 25 functional categories. In the second step, we calculated distance matrices between the genomes on the basis of their orthogroup count profiles constructed by OrthoFinder as described above. We did this separately for each functional category and then visualized the distance matrix of each functional category by performing a PCoA of each of them.

### Interest-driven approaches.

Screening for catalase- and SOD-encoding genes was done by three different methods. First, the presence of the gene of interest was evaluated on the basis of the Prokka annotation. Second, known variants of genes of interest were manually identified from the literature (NCBI database gene accession numbers 1062016, 29637976, and 4413348 for catalase; NCBI database nucleotide accession number HM070825 for SOD) and then blast searched against the pangenome. Third, the Pfam database was used to download HMMs of the protein families of the genes of interest (PF00199 and PF05067 for catalase; PF00080, PF00081, PF02777, and PF09055 for SOD). HMMER (22) was then used to scan the pangenome against these HMMs. GTs were detected by screening a database of orthogroup representative sequences against profile HMMs downloaded from dbCAN (54) supplemented with three Pfam profile HMMs (PF02397, PF02109, and PF04756) with HMMER with an E value cutoff of 1e-18. Further processing and visualization were done with custom R scripts.

### Data availability.

Raw sequence data and assembled contigs of AMBR2 are available at the European Nucleotide Archive under accession number PRJEB21025. All of the scripts used in this study are available at https://github.com/LebeerLab/caseiGroup_mSystems_pipeline. The genome of *L. casei* AMBR2 has been deposited in the Belgian Coordinated Collections of Microorganisms under accession number LMG P-30039.
