# Supplementary material for: Large-Scale Phylogenomics of the Lactobacillus casei Group Highlights Taxonomic Inconsistencies and Reveals Novel Clade-Associated Features
Source: mSystems. 2017 Aug 22;2(4):e00061-17. doi: 10.1128/mSystems.00061-17 (PMC5566788; doi:10.1128/mSystems.00061-17)
Supplement: TABLE S2 [file sys004172127st2.docx]

|  |  | AN | AN+ | AE | AE+ |
| --- | --- | --- | --- | --- | --- |
| Clade A | L. casei ATCC334 | - | - | - | - |
|  | L. casei LcA | - | - | - | - |
|  | L. casei LcY | - | - | - | - |
|  | L. paracasei JCM 8130 | - | - | - | - |
| Clade B | L. casei AMBR2 | ++ | ++ | ++ | ++ |
|  | *L. zeae* DSM20178 = KCTC 3804 | - | + | - | - |
|  | L. casei DSM20011 | - | + | - | ++ |
|  | L. casei subsp. casei ATCC393 | - | + | - | + |
| Clade C | L. rhamnosus GG | - | - | - | - |
|  | L. rhamnosus Lc 705 | - | - | - | - |
|  | L. rhamnosus DSM 20021 | - | - | - | - |
|  | L. rhamnosus GR1 | - | - | - | - |

# References

1. Zotta T, Ricciardi A, Ianniello RG, Parente E, Reale A, Rossi F, Iacumin L, Comi G, Coppola R. 2014. Assessment of aerobic and respiratory growth in the Lactobacillus casei group. PLoS One 9. https://doi.org/10.1371/journal.pone.0099189.

2. Zotta T, Ricciardi A, Guidone A, Sacco M, Muscariello L, Mazzeo MF, Cacace G, Parente E. 2012. Inactivation of *ccpA* and aeration affect growth, metabolite production and stress tolerance in *Lactobacillus plantarum* WCFS1. Int J Food Microbiol 155:51–59. https://doi.org/10.1016/j.ijfoodmicro.2012.01.017.
